# Gold-Shell Crowns One Hundred and Fifty Years Ago

**Published:** 1897-10

**Authors:** William H. Trueman

**Affiliations:** Philadelphia


### GOLD-SHELL CROWNS ONE HUNDRED AND FIFTY YEARS AGO.

BY DR. WILLIAM H. TRUEMAN, PHILADELPHIA.

A French work, entitled "Essay d'Odontotechnie, ou Dissertation sur les Dents Artificielles," written by M. Mouton, a surgeon-dentist of Paris, and there published in 1746, is, so far as I now know, the first work written exclusively upon prosthetic dentistry. In it the writer describes and advises the use of the device we now know as a gold-shell crown,—a cap of gold covering, and made to accurately fit the whole exterior of a tooth whose usefulness has been impaired by loss of substance. He especially advises and urges their use upon the molars. He notes that objection may be made to the gold being conspicuous when they are placed upon teeth farther front, and states that when there are no other objections to their use upon these conspicuous teeth than the color, he overcomes this by enamelling the front of the cap, making it the same color as the adjoining teeth.

The work is not technical, but is written for popular reading in an instructive and entertaining style, and, while not entering into constructive details, out of place in a work of this character, it gives a fair insight of the art of prosthetic dentistry as it then existed. To the operative branch he refers only incidentally. He earnestly advocates saving to the utmost the natural teeth, and to this end strongly urges frequent and regular visits to the dentist, cleanliness, the prompt and thorough removal of deposits, and careful attention to all cavities of decay as soon as they appear. Among the materials he uses in this art, the only metal named is gold. This he uses in the form of wire for attaching false teeth to natural teeth or roots; in the form of leaves, as he expresses it, "pour en remplir les cavités faites par la carie." The word "remplir," as here used, means "to fill," "to cram," etc., and no doubt from it we get our expressions "plug" and "plugging," "fill" and "filling." He also uses gold in plate, or thin lamina, for regulating. For the benefit of those who may be interested, I copy from the original his remarks upon gold caps. It is old style, f being used for s. On page 137 he says,—

"Il faut recouvrir la Dent ufée d'une calotte d'or, qui incrufte toute fa furface extérieure, et qui foit ajuftée de maniere que elle ne puiffe intercepter aucune portion d'alimens. La Dent oppofée, et les alimens n'ayant plus alors d'action fur le corps de la Dent ainfi revêtu, elle eft préfervée, quelque tendre qu'elle puiffe être, du dommage dont nous parlons.

"Cette pratique eft très-avantageuse pour les groffes Dents, ou Molaires, attendu qu'elles caufent beaucoup de douleur, quand la Dent commence à s'ufer près du nerf, et qu'il n'y a d'autre moyen pour l'arretêr, que celui de facrifier la Dent. On eft fûr de fa confervation, fans craindre qu'elle caufe dans la fuite aucun mal, lorfque l'on s'y prend de bonne heure et à tems, pour la faire recouvrir. L'inconvénient que l'on peut trouver à faire la même opération aux petites Molaires et aux incifives, eft qu'étant placées au-devant de la bouche, elles font toujours expofées à la vûe par les divers mouvemens que les lévres font, foit en parlant, foit en riant. Les yeux, fans doubte, feroient choqués d'une couleur auffi difparate, que celle de l'enveloppe que je propofe pour les Molaires; mais lorfque la difpofition des incifives ne s'oppofe pas à cette reffources, l'on peut faire émailler l'extérieur de l'enveloppe de la même couleur que les Dents voifines."

